# *Staphylococcus aureus* Exotoxins and Their Detection in the Dairy Industry and Mastitis

**DOI:** 10.3390/toxins12090537

**Published:** 2020-08-20

**Authors:** Ana G. Abril, Tomás G. Villa, Jorge Barros-Velázquez, Benito Cañas, Angeles Sánchez-Pérez, Pilar Calo-Mata, Mónica Carrera

**Affiliations:** 1 Department of Microbiology and Parasitology, Faculty of Pharmacy, University of Santiago de Compostela, 15898 Santiago de Compostela, Spain; anagonzalezabril@hotmail.com; 2 Department of Analytical Chemistry, Nutrition and Food Science, School of Veterinary Sciences, University of Santiago de Compostela, 27002 Lugo, Spain; jorge.barros@usc.es (J.B.-V.); p.calo.mata@usc.es (P.C.-M.); 3 Department of Analytical Chemistry, Complutense University of Madrid, 28040 Madrid, Spain; bcanas@quim.ucm.es; 4 Sydney School of Veterinary Science, Faculty of Science, University of Sydney, Sydney, NSW 2006, Australia; angelines2085@icloud.com; 5 Department of Food Technology, Spanish National Research Council (CSIC), Marine Research Institute (IIM), 36208 Vigo, Spain

**Keywords:** *Staphylococcus aureus*, exotoxins, mastitis, *Staphylococcus aureus* detection, staphylococcal food poisoning, food-borne pathogen

## Abstract

*Staphylococcus aureus* constitutes a major food-borne pathogen, as well as one of the main causative agents of mastitis in dairy ruminants. This pathogen can produce a variety of extracellular toxins; these include the shock syndrome toxin 1 (TSST-1), exfoliative toxins, staphylococcal enterotoxins (SE), hemolysins, and leukocidins. *S. aureus* expresses many virulence proteins, involved in evading the host defenses, hence facilitating microbial colonization of the mammary glands of the animals. In addition, *S. aureus* exotoxins play a role in the development of both skin infections and mastitis. Indeed, if these toxins remain in dairy products for human consumption, they can cause staphylococcal food poisoning (SFP) outbreaks. As a result, there is a need for procedures to identify the presence of exotoxins in human food, and the methods used must be fast, sensitive, reliable, and accurate. It is also essential to determine the best medical therapy for human patients suffering from *S. aureus* infections, as well as establishing the relevant veterinary treatment for infected ruminants, to avoid economic losses in the dairy industry. This review summarizes the role of *S. aureus* toxins in the development of mastitis in ruminants, their negative effects in the food and dairy industries, and the different methods used for the identification of these toxins in food destined for human consumption.

## 1. Introduction

*Staphylococcus aureus* is a frequent contaminant of foodstuffs, and possibly represents the main food-borne pathogen that causes health problems in both animals and humans. In addition, it is one of the major causes of bovine, ovine and caprine mastitis, resulting in substantial reduction in both milk production and quality, generating considerable economic losses in the dairy industry [1]. *S. aureus* is a Gram-positive catalase, coagulase, and usually Voges-Proskauer positive organism. It is a non-spore-forming, oxidase-negative, nonmotile, cluster-forming, and facultatively anaerobic microorganism that grows at a wide range of temperatures and pHs. In humans, *S. aureus* colonizes the nasal mucosa and skin in an estimated 50% of the healthy population, but it can also cause hematological infections [2]. *S. aureus* was firstly identified in 1884 and the mortality rate for patients with *S. aureus* bacteremia was 82%. With the introduction of penicillin, the mortality rate was largely reduced. In the 1940s, the first penicillin-resistant strain appeared and in the 1960s, only one year after the introduction of methicillin and oxacillin, methicillin-resistant *S. aureus* (MRSA) strains emerged. In the 1990s, the percentage of infections related to MRSA was 29% [3]. On the other hand, it is known that in countries such as China and others, the 10–40% of mastitis cases are caused by *S. aureus*; and more than 90% of *S. aureus* food poisoning outbreaks are related to staphylococcal enterotoxins (SEs) [4]. This bacterial pathogen can produce a variety of extracellular proteins, known as exotoxins, that include enterotoxins, hemolysins, and leukocidins. The latter destroy white blood cells, while enterotoxins can stimulate the host T cells and cause a cytokine storm [5]. In milk-producing ruminants, *S. aureus* is normally the major cause of mammary gland inflammation, referred to as mastitis [6]. Although *S. aureus* is responsible for 95% of the coagulase positive tests in bovine-mastitis samples, this test is not sufficient to identify the bacterium, requiring additional diagnostic confirmation. The incidence of *S. aureus* mastitis can be reduced with hygienic measures and habits, as well as with good management of milking practices. These additional measures are essential in the management of dairy goat and sheep, as their milk and derivative products are often not subjected to heat treatments such as pasteurization; hence, human consumption of these foodstuffs can result in food poisoning [1].

*S. aureus* exotoxins play a role in skin infections, including mastitis, and, as a result, they can be present in dairy products; human consumption of these foodstuffs can result in food poisoning (SFP) outbreaks. The fast, sensitive, and accurate detection of exotoxins in mammary glands, and indeed in food, is essential, both to identify the best treatments for *S. aureus* infections and to prevent economic loss in the dairy industry. This review summarizes the role of *S. aureus* toxins in mastitis outbreaks, their effect on the food and dairy industries, and finally, the use of different techniques to identify these toxins in foodstuffs, to avoid food poisoning.

## 2. Role of *Staphylococcus aureus* Toxic Proteins in Mastitis

Skin is the mayor physical and immunologic barrier to bacterial infection, and some of the epidermal cells, such as keratinocytes, express recognition receptors after contact with microorganisms; the skin produces antimicrobial peptides, that constitute an early cutaneous immune response [7]. Additional immune cells present in the skin include Langerhans and dendritic cells, macrophages, mast and plasma cells, natural killer cells, and T and B cells. Commensal organisms present in the skin, such as *Staphylococcus epidermidis* and *Propionibacterium acnes*, provide an additional line of defense, preventing the growth of pathogens [8]. However, *S. aureus* pathogenicity involves the production of virulence factors, that help the bacterium evade host defenses, hence allowing the microorganism to colonize the mammary glands of ruminants. These factors include structural components (collagen, fibrinogen, elastin binding proteins, penicillin binding protein, teichoic acid, protein α, β-lactamase, proteases, capsules, and slimes), enzymes (coagulase, staphylokinase, DNase, phosphatase, lipase, phospholipase, hyaluronidase), and toxins (staphylococcal enterotoxins, toxic shock syndrome toxin 1, leukocidin, hemolysins, and exfoliatin). In addition, *S. aureus* has specific mechanisms, such as biofilm formation, that facilitate its adherence to and invasion of mammary epithelial cells.

**Figure 1:**
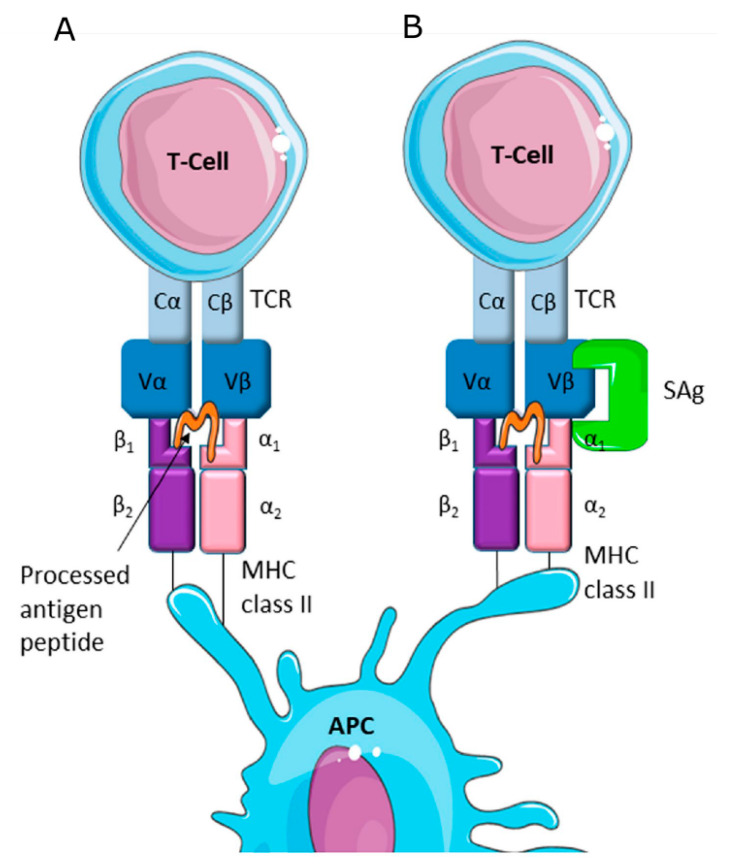
Mechanisms of T-cell activation: (**A**) Conventional T-cell activation by antigen presenting cells (APC) and (**B**) the association of three proteins, *S. aureus* Sags, MHC class II, and the TCR β-chain, act as an unconventional activation complex, that triggers uncontrolled activation of T-cells. Image from Tuffs et al., 2018 [3], under Creative Common License.

*S. aureus* enterotoxins include toxic shock syndrome toxin 1 (TSST-1), exfoliative toxins, and staphylococcal enterotoxins (SEs) (Table 1). Exfoliative toxins (ETs) are serine proteases with affinity towards desmoglein 1 (Dsg1) that help *S. aureus* colonize the skin of mammals [6]. Hemolysins, such as β, α, and δ toxins (Table 1), attack cell membranes and cause platelet damage, lysosome destruction, ischemia, and necrosis. Hla (α hemolysin) is a beta-barrel pore-forming toxin that disrupts the cell membrane causing irreversible osmotic changes, resulting in cell-death by apoptosis. Hla can damage the membrane of a variety of cells, such as lymphocytes, red blood cells, platelets, and endothelial cells. Hla binds to the target cell via its cellular receptor, the transmembrane protein ADAM10, creating a heptameter of β-barrels that extrudes the lipid bilayer and forms a transmembrane channel. This leads to cell permeability, resulting in cell death and creating a pro-inflammatory stimulus [9]. Beta hemolysin (Hlb) is encoded by a lysogenic bacteriophage; in itself, it cannot destroy most cell types, but it exposes vulnerable cells to other lytic proteins, such as Hla and leukocidins [10]. This toxin, also known as sphingomyelinase, displays high hemolytic activity against sheep erythrocytes; it can also damage keratinocytes, helping the bacterium colonize mammalian skin. The different susceptibility of erythrocytes from different species to Hlb may be due to the amount of sphingomyelin present in the cells [9]. Leukocidins are also pore-forming two-component toxins that specifically attack immune cells. There are seven different types of leukocidins, and the Panton-Valentine leukocidin (PVL) is the toxin with the strongest effect on immune cells; additional cytotoxins, such as LukPQ and LukMF’, are also important, but are only effective against a limited host species range, including ruminants. SEs and TSST-1 are pyrogenic toxins known as staphylococcal superantigens (SAgs), that interact with components of the major histocompatibility complex class II (MHC-II). SAgs display two major domains, an oligosaccharide/oligonucleotide binding (OB) fold at the N-terminal domain, and a C-terminal domain with a β-grasp topology. SAgs binding to MHC-II induce activation, in a T cell receptor (TCR) Vβ-specific manner, cellular proliferation, and production of large amounts of cytokines [2]. The association of SAgs, MHC class II, and the TCR β-chain constitute an unconventional T cell activation complex provoking a massive T cell response [3] (Figure 1). Furthermore, the first community-associated methicillin-resistant *S. aureus* (CA-MRSA) strain described contained acquired pathogenicity islands (SaPIs) encompassing new exotoxin genes [5]. In addition, mobile genetic elements can contain enterotoxin genes; these elements include plasmids, prophages, and genetic cassettes, such as the staphylococcal cassette chromosome (SCC) and the enterotoxin gene clusters (egc) [11]. Genome sequencing of a variety of *S. aureus* strains demonstrated that some exotoxin genes are placed in clusters within the pathogenicity islands or the υSa; they include leukocidins, TSST-1, and SEs. The exotoxin gene cluster (egc), that contains five SE genes (*seg*, *sei*, *seo*, *sen*, and *sem*) and two pseudogenes, is also part of a genomic island. It is important to note that a single *S. aureus* strain can carry more than one SaPI [12].

**Figure 2:**
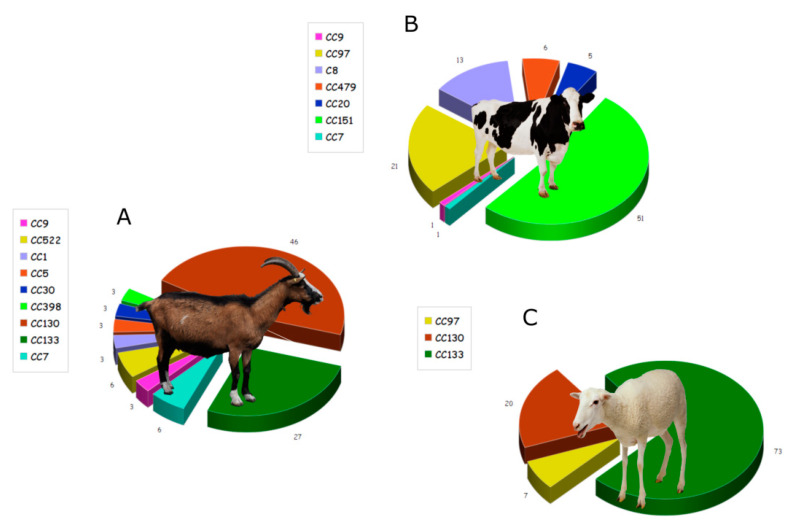
Distribution of clonal complexes (CC) in *S. aureus* strains isolated from the milk of different ruminant species: (**A**) goat (*n* = 34), (**B**) cow (*n* = 78), and (**C**) sheep (*n* = 15). Image modified from Merz et al., 2016 [1], under Creative Commons Attribution License (CC BY).

*S. aureus* can also produce small colony variants (SCV), that are better adapted to survive in mammalian cells, as they are less affected by host defense mechanisms [13]; SCV are resistant to aminoglycoside antibiotics and can cause recurrent infections. However, their phenotype is not stable and mutations usually revert to a normal phenotype [14]. Naturally occurring *S. aureus* strains have a high genetic diversity; a variety of virulence factors have been found in staphylococcal isolates from bovine intramammary infections. Comparison of *S. aureus* genotypes isolated from either bovine, ovine, or caprine animals suffering from mastitis suggests the existence of specific *S. aureus* clonal types for each of the animal groups; the bacterium appears to have developed distinct virulence mechanisms to specifically target these three ruminant groups. These studies centered on the prevalence of individual toxins, with the aim of understanding the role of each particular toxin in the development of mastitis [12,13,15,16,17,18,19,20,21,22,23]. Indeed, Vaughn and coworkers [13] studied the genotypes of individual *S. aureus* isolates, identified from cases of bovine mastitis in east Tennessee. They described a variety of diverse genotypes that, although containing a variety of enterotoxin genes, displayed a high prevalence for *seb* (SEB gene) and *tsst-1*. Merz and collaborators determined the profiles of virulence and resistance genes, including toxins, as well as the clonal complexes (CC) in *S. aureus* isolated from either goat, cow, or sheep milk (Figure 2).

This study reached the expected conclusion that, although *S. aureus* isolates from goat and sheep milk were closely related, both were different from the bacteria present in bovine milk [1]. Different pathogenic properties require different detection methods, both for disease treatment and for gene identification [2]. To successfully treat this pathogen, it is essential to identify and characterize the virulence gene profiles and the clonal diversity present in *S. aureus* isolates [24].

*S. aureus* strains associated with bovine mastitis harbor a variety of staphylococcal SAg toxin genes; these are normally acquired by horizontal gene transfer via genetic mobile elements or bacteriophages, and even by bacterial mating. Some studies have determined the relationship between SAg toxin genes, genotypes, and pathogenic properties of *S. aureus* isolates, by methods such as randomly amplified polymorphic DNA (RAPD) [2], pulsed-field gel electrophoresis (PFGE), reversed passive latex agglutination (RPLA), and PCR [25,26,27,28,29]. Staphylococcal enterotoxins G to Q (SEG–SeEQ) are the most frequently found in *S. aureus* strains isolated from bovine mastitis; these enterotoxins are involved in the mammary inflammatory present in the animals. However, staphylococcal enterotoxin expression is influenced by regulatory elements that respond to environmental stimuli, hence, the concentration of the toxins is different when the bacteria are grown under laboratory conditions.

Moreover, many researchers recommend the use of anti-virulence compounds that target a variety of virulence factors, as well as using a combination of these compounds; this, in turn, could be more effective than conventional treatments [24]. These therapies work by interfering with the virulence factors and bacterial toxins; besides, they can interfere by regulating their production pathways. As an example, the *agr* gene, which is involved in *S. aureus* α-, β-, and γ-hemolysins, TSST-1, SEB, SED, and SEC, exfoliatin A and B and PVL regulation, and which has been extensively studied. Moreover, other regulatory systems such as the Staphylococcal accessory regulator A (SarA) *S. aureus* exoprotein expression (Sae) operon, and the Staphylococcal alternative sigma factor B (SigB) have also been found to control the production of virulence factors and toxins [30]. Fang and collaborators applied purified staphylococcal enterotoxin C to murine mammary glands and demonstrated that the treatment produces a significant increase in proinflammatory cytokines, as compared to the untreated controls [17]. Furthermore, mice treatment with anti-staphylococcal enterotoxin C antibodies, significantly reduced tissue damage and mammary gland inflammation; this confirms, which means that staphylococcal enterotoxin C is involved in mastitis development [5]. However, it is not yet known how enterotoxins contribute to the development of mastitis [13], although some reports indicate that the superantigen toxins can diminish the host immune response [16].

Furthermore, a variety of leukocidins were identified in *S. aureus* species, including the phage-encoded PVL, LukPQ, and LukMF’ toxins, which can affect the immune system, although these toxins display a limited host range. LukMF’ is encoded by prophages that lysogenize *S. aureus* strains of ruminant origin; this protein, although a potent killer of bovine neutrophils, macrophages, and monocytes, does not have any activity on human neutrophils [17]. *S. aureus* pore-forming toxins (PFTs), α-toxin and leukocidins, were discovered approximately one century ago, but their host cell specificities have only just been deciphered in the last seven years; these findings highlight the specificity of toxins and their binding sites, as far as mammal species and cell types are concerned [31]. Leukocidin LukMF’, a putative of bovine neutrophils killer in vitro, displays its highest activity on bovine neutrophils [32]. This protein binds to the CCR1 receptor on bovine neutrophils, which is absent on human neutrophils. Hoekstra et al. [17] studied the role of LukMF’ in natural infections, demonstrating that the genes encoding this protein were present in 96% of *S. aureus* isolates obtained from mastitis milk samples. Moreover, bacterial isolates that produce high levels of LukMF’ contain a point mutation in the repressor of toxins (*rot*) gene; this blocks Rot protein translation and increases LukMF’ production. Rot can bind to the promotor region of toxin genes, such as *hla*, *hlgC-hlgB*, *lukE-lukD*, and *lukA-lukB*, resulting in a non-functional start codon, hence acting as a global regulator of *S. aureus* virulence gene expression. In addition, leukocidin expression (LukAB, LukED, PVL) increases when the *rot* gene is not active [17].

In addition, *S. aureus* hemolysins are involved in the development of the mastitis infection. Hlb damages mammary epithelial cells, improving *S. aureus* adhesion and bacterial multiplication, while Hla increases the damaging effects by facilitating cell lysis [10]. The diversity among both *hla* gene sequences and the clonal profiles of *S. aureus* strains isolated from bovine mastitis were determined in order to evaluate their inter-relationships. *S. aureus* isolates display different patterns of multi-drug resistance, while the *hla* gene also exhibits high diversity (14 genotypes); however, Hla peptides are relatively more conserved. Interestingly, based on phylogenetic analysis, it was possible to establish that there is a distinct evolutionary relationship between *hla* genes and MLSTs (multilocus sequence types) [24]. A similar study demonstrated that the distribution of hemolysin phenotypes does not correlate with the hemolysin genes present in *S. aureus* strains isolated from mastitis in dairy cows; in an apparent contradiction, α-hemolysis, not β-hemolysis, was the dominate hemolytic phenotype encountered, even though the *hla* gene was identified in less bacterial isolates than the *hlb* gene [10]. These findings were further confirmed by the fact that Hla antisera inhibited the toxic effects of *S. aureus*, by reducing adhesion to the bovine mammary epithelial cell layer. Hla induces beneficial immunogenicity in *S. aureus* infection, while Hla vaccination can increase neutrophil numbers and reduce the amount of bacteria present in the milk [10]. Furthermore, vaccination of ewes with partially purified Panton-Valentine leukocidin, contaminated with α-hemolysin, was described to produce partial protection against mastitis-causing *S. aureus* strains [19].

Exfoliative toxins (ETs), which hydrolyze desmoglein 1 (Dsg1), causes dissociation of keratinocytes in both human and animal skin. Three different ETs (ETA, ETB, and ETD) are associated with skin exfoliation in different hosts, but with varied effects, indicating at least some degree of host specificity; a caveat is that this specificity was only studied in bacterial strains associated with mild mastitis in ewes. A new exfoliative toxin, ETE, was recently identified from sheep suffering from mastitis caused by *S. aureus* O46; the implication is that ETE could contribute to bacterial colonization of stratified epithelia [6].

A variety of global studies have analyzed the production of different *S. aureus* toxins, that could play a role the development of mastitis [12,33]. A total of 84 different *S. aureus* strains were isolated from the milk of cows with subclinical mastitis and analyzed. These bacteria were tested, by classical PCR and multiplex PCR, for the presence of a variety of hemolysins (*hla*, *hlb*, *hld*, *hlg*, and *hlg-2*), leukotoxins (*lukPV*, *lukM*, and *lukED*), toxic shock syndrome toxins (*tst*), and enterotoxins (*sea* to *see*, *seg* to *ser*, and *seu*) genes. The results obtained allowed the classification of the exotoxin genes discovered into 14 profiles, or EGs; they also revealed that most of the SAg genes were located on two pathogenicity islands. This indicates that there are a wide variety of toxigenic *S. aureus* genomic types that are both causal agents of clinical diseases and risk factors for the food poisoning mentioned above [12].

## 3. *Staphylococcus aureus* Exotoxins in Food

*S. aureus* can grow in a variety of foodstuffs, such as raw milk and dairy products, which constitute good substrates for *S. aureus* contamination. This means that there are many paths by which the pathogen can enter into dairy food destined for human consumption; this can be achieved either through the people employed in the dairy industry, the environment and milking equipment, or directly from the animals, as it can be found in dirty udders and surrounding skin. One important additional possible source of milk contamination is when the dairy animals (either goats, cattle, or sheep) suffer from *S. aureus*-induced mastitis [34]. As mentioned above, *S. aureus* can cause food poisoning (SFP) outbreaks, by ingestion of staphylococcal enterotoxins (SEs); the common symptoms of these poisonings are vomiting, diarrhea, nausea, and abdominal cramps, which appear 2–6 h after SE-contaminated food consumption. Just a few micrograms of SEs are sufficient to cause SFP in vulnerable adults, while 100 ng are enough in children [34]. SEs poisoning results from an inflammatory response that causes serious damage in the jejunum and ileum and alters the expression of cytokines, the production of T cell metabolites, macrophages, monocytes, and mastocytes [3,34]. The enterotoxins, in conjunction with TSST-1, can act as superantigens (SAgs). SAgs bind to both the Vβ chain of T-cell receptors and to MHC class II molecules on antigen presenting cells, which, in turn, activates additional T-cells, resulting in a massive cytokine secretion, inflammation, and toxic shock syndrome (TSS) [11].

To date, 23 staphylococcal enterotoxins have been identified, based on their antigenicity. Staphylococcal enterotoxins SEA, SEB, SEC, SED, and SEE are well characterized, they are approximately 220–240 amino acids long and display similar molecular weights (25–30 kDa); it is important to note that commercial kits are currently available for the detection of these toxins. Typically, *S. aureus* SE food poisoning is associated with inappropriate handling of cooked foodstuffs, or improper storage, resulting in bacterial growth and toxin production [11]. These enterotoxins are mainly found in foods with high amounts of starch and protein, such as meat products, eggs, and dairy products [35,36]; but enterotoxin production is also affected by other factors, such as pH, water activity, temperature. The SEA, SEB, SEC, SED, and SEE classic enterotoxins are responsible for 95% of SFP; with SEA and SED as the most common causes of food poisoning, due to their stability under a wide range of water activities and pH conditions [34,37]. Recent published papers identified additional SEs (SEG, SEH, SEI, SER, SES, SEIY, and SET) as putative food poisoning agents. This group of proteins represent SE-like toxins (SEls), due to the fact that they either lack emetic properties or have yet to be tested [11,38].

Toxic shock syndrome (TSS) is caused by TSS-1, which is a potent toxin occuring in 0.006 cases per 100,000 people. TSS is characterized by hypertension, rash, fever, constitutional symptoms, and multi-organ failure, finally resulting in death. As is the case for SAg, TSS-1 activates T cells by increasing immunological activity. The presence of TSS-1, in either humans, animals, or food, is associated with food poisoning, but its prevalence is not yet as well-known as SEs [37]. The TSST-1 toxin load has been studied in different types of milk under different storage conditions (pasteurized and UHT milks treated at 15 and 22 °C), as well as following inoculation with different amounts of *S. aureus*. The results indicated that, while unpasteurized milk harbored the highest bacterial content, as expected, pasteurized milk displayed a higher TSS-1 load. This is probably a reflection of the presence of lactic acid bacteria in pasteurized milk, which is known to have a down regulatory effect on TSS-1 gene expression.

It remains to be established why the two SAgs toxins, SE and TSST-1, display different pathogenesis in food poisoning caused by *S. aureus*. Although both toxins can be produced at the same time, their expression levels are different, resulting in different toxic syndromes. SEA isoforms are highly stable, withstanding the activity of most proteolytic enzymes, such as pepsin, trypsin, chymotrypsin, rennin, and papain. Staphylococcal SEB can be inactivated by pepsin digestion, at pH 2.0, although it is pepsin-resistant at higher pH values [39]. TSST-1 is stable under a variety of negative conditions, such as heat treatment, pepsin and trypsin digestion; in fact, it retains its superantigen activity even when degraded to smaller peptides [37].

SEs are low molecular weight proteins that are resistant to extreme conditions, such as low pH, freezing, and drying. Heat-treatment of foodstuffs causes the death of *S. aureus*, but TSST-1 and enterotoxins survive the treatment, remaining active even in the digestive tract after ingestion. These SEs are sometimes not detected by methods such as serology [5]; due to the importance of these toxins in the public health and food sectors, there is an urgent need to develop techniques that allow a fast, sensitive, and accurate detection and identification of these toxins, and their prevalence [34].

## 4. *Staphylococcus aureus* Exotoxin Identification in Food and Milk

The presence of either viable *S. aureus* cells or toxins in foodstuffs is directly related to poor sanitation procedures. Human food is routinely treated to eliminate harmful bacteria, but many toxins are stable under heat treatment, even when carried out over extended periods of time. Detection of harmful substances, such as *S. aureus* toxins, in foodstuffs is of particular importance, but current laboratory procedures, to detect *S. aureus* contamination in samples, such as food or blood, are time-consuming and require special resources. On the other hand, many novel methods have been described, that allow faster pathogen identification and, hence prompt therapeutic intervention; in addition to the fact that there are currently a variety commercial assay kits, for both the detection of viable *S. aureus* cells and toxin identification. Table 2 summarizes a selection of the commercial kits available, with a special focus on enterotoxin identification in foodstuffs [8].

In recent years, the development of molecular methods has revolutionized pathogen identification, including both *S. aureus* and the toxins it produces. For instance, *S. aureus* toxin identification can be carried out by either Western blot, radio immunoassay, enzyme-linked immunosorbent assay (ELISAs), or by the use of aptamers and PCR techniques. Additional sensitive methods, such as immuno-PCRs, mass spectrometric analysis, reversed passive latex agglutination assays, and biosensor techniques also allow detection and quantification of exotoxins, such as α-hemolysin, enterotoxins, and PVL toxins, in foodstuffs. Many procedures permit the simultaneous identification of a variety of toxins, such as SE, TSST-1 [18,22], and exfoliative enterotoxins [40]; although detection of *S. aureus* exotoxins in food is mainly restricted to enterotoxins, and occasionally TSST-1 toxin. Identification of additional exotoxins, such as PVL, is usually carried out in clinics, and involves techniques such as PCR, ELISA, and immunochromatographic assays [41,42,43,44]; this is also the case for exfoliative toxins [45] and hemolysins [46]. It must be taken into account that the diagnosis based on the study of toxin genes could lead to false-positive results, since the gene can be present in the sample, but without expression. Looking directly for the toxins by a variety of techniques can be more appropriate, thus avoiding the false-positive results. Each employed technique shows different difficulties and time-consuming procedures. In the case of immunoassays and molecular gene-based methods, they can be applied directly to the food sample or with a previous toxin or nucleic acid extraction and purification, respectively [11]. On the other hand, for mass spectrometric techniques, the toxins should be extracted and purified from the samples before passing through the columns to obtain the best possible result [34,47,48]. Moreover, the food matrix and the need for specialized personnel must be taken into account when selecting the method for toxin detection.

### 4.1. Staphylococcus aureus Toxin Identification Using Conventional Methods

SE tests were originally used to detect toxins in infected animals, such as kittens, guinea pigs, and monkeys; and the physiological changes, such as vomiting and diarrhea, were subsequently studied. These tests have the drawback of displaying low sensitivity and specificity [34].

Moreover, serological tests based on antigens/antibodies represent some of the earliest methods implemented to detect SEs; the techniques utilized included diffusion and agglutination tests. Some of these assays involved the analysis of SEB in foodstuffs, such as custard, chicken, and shrimp. A double-gel diffusion test developed could detect 0.002 µg of SEA and 0.05 µg of SEB per gram of cheese. The latex agglutination inhibition test, developed later, was reported to detect 0.5 mg/mL of SEB in liquid food. After that, reverse passive latex agglutination (RPLA) tests allowed SE detection in pasta, beef, ham, cooked turkey, and some dairy products [34]. Further assays, such as the traditional gel diffusion method and a variety of RFLA-based kits (SET-RPLA and TST-RPLA) were used to detect SEs and toxic shock TSST-1 toxin production in a variety of clinical samples, like those originating from food poisoning outbreaks [49]. RPLA techniques were also developed for PVL detection [50]; RPLA uses latex particles coated with enterotoxin antibodies that agglutinate in the presence of enterotoxins. These methods were eventually superseded, due to their limitations, they are semi-quantitative and display low sensitivity and specificity; in addition, the foodstuffs to be analyzed must be extremely clean, to avoid false-coagulation reactions [39].

### 4.2. Staphylococcus aureus Toxin Identification Using Genomic Methods

Nucleic acid hybridization techniques, such as colony blot hybridization, allow not only the identification of different enterotoxin genes, but also of different toxigenic *S. aureus* isolates. Dot blot hybridization has been used, for some time now, to detect SE genes in staphylococcal strains in laboratories; while gene amplification, by PCR, is widely used to detect SEs, such as toxin genes *sea–see* and *seg–seq*, and the toxic shock syndrome gene *tsst-1* [51]. These genes in *S. aureus* isolates were also analyzed by pulsed-field gel electrophoresis (PFGE), using the restriction endonuclease SmaI [18]. The *entB* and *entC1* genes, encoding the SEB and SEC proteins respectively, were the first genes amplified by PCR in dried skim milk powder; while dairy products, meat, and traditional sweets have been analyzed using multiplex PCR to detect the *S. aureus* 16s rRNA, as well as the *sea*, *seb*, *sec*, *sed*, and *see* genes [52]. Several studies have used multiplex PCR to amplify a variety of toxin genes, in combination with genes associated with biofilm formation, such as *icaA* and *icaD* [20], clumping factor (*clfA*), or protein A (*spa*) [15]; these co-amplifications helped establish the relationships between those genes, as well as furthering research into their role in staphylococcal infections. In addition, both PCR and ELISA techniques have been successfully used to investigate the presence of a variety of genes in beef and lamb meat; these genes include those corresponding to SE (*sea*, *seb*, *sec*, *sed*, and *see*), several SEI toxins (*seg*, *seh*, *sei*, *sej*, *sel*, *sem*, and *seo*), exfoliative toxins (*eta* and *etb*), and the toxic shock syndrome 1 toxin (tst) [40]. Apart from multiplex-PCR [53], other PCR techniques developed, such as real-time PCR, have allowed the detection of the enterotoxin gene cluster (*egc*) in raw milk [54]. Additional PCR technologies, such as reverse-transcriptase PCR (RT-PCR), immunocapture polymerase chain reaction (IC-PCR), and PCR-enzyme linked immunosorbent assay (PCR-ELISA) have also proved to be successful in the detection of toxins with a sensitivity in the range of 10 pg/mL [55,56]. Furthermore, PCR-EIAs have been developed for the detection of *S. aureus entA*, *entB*, *entC*, *entD*, and *entE* genes; while additional PCR-EIAs have also been developed to detect the staphylococcal exfoliative toxin genes (*eta* and *etb*) and the toxic shock syndrome toxin gene *tst*. These two technologies not only provide fast, sensitive, and specific results, but they also allow simultaneous detection of the genes [57].

In summary, PCR-based methods can be applied to successfully detect SEs in a great variety of foods; however, false negative results can occur, due to interference from contaminating products obtained from the target cells during procedures such as nucleic acid extractions, that can either cause target nucleic acid degradation or direct inhibition of the DNA polymerizing enzyme [34]. Alternative nucleic acid-based methods, such as NASBA (nucleic acid sequence-based amplification) and LAMP (loop-mediated isothermal amplification), can also be used for the detection of foodborne pathogens and their toxins; these methods do not have the caveat of requiring a thermocycler instrument, while still being specific, sensitive, and cost efficient [54].

### 4.3. Staphylococcus aureus Toxin Identification Using Immunoassays

Antigen/antibody methods have a wide application in many fields, and can also be used to detect SEs in complex samples without extensive pretreatment. These immunoassay methods include ELISA, enzyme-linked fluoroimmunoassay (ELFA), and immunomagnetic separation, using beads [56]. Direct ELISA and sandwich ELISA, in that order, displayed the highest accuracies for toxin detection in milk [58]; with immunoassays having the advantage of not requiring purified protein samples, allowing SEs detection in samples taken directly from culture or those contaminated with other food products. VIDAS, TRANSIA, TECRA, and RIDASCREEN represent commercially available test kits, using the sandwich ELISA technology for the detection of SEs (Table 1) [8]; with VIDAS allowing SE detection in both milk and cheese [54].

Biosensors are instruments that recognize biological or chemical metabolites, they produce either an optical or electrical signal with an intensity that is proportional to the amount detected, hence allowing metabolite quantification. They are composed by a biomacromolecule, the element responsible for metabolite recognition, and a transducer, that generates the signal [59]. There are three principal types of sensors, responsible for either optical, electrochemical, or mass detection techniques. These technologies are easy to implement, and biosensors have been used to detect SEs in either foodstuffs, the environment, or in other substrates; their detection limits are 8.7 ng/mL for SEA and 6 ng/mL for SEC [43].

Immunosensors are devices that combine immunoassays with biosensors, they are highly sensitive and specific and can recognize the antibody signals and generate measurable transduction patterns [34]. These advantages have made them popular in recent years, and optical-based immunoassays are widely used for SE detection in foodstuffs. Moreover, a variety of ELISA techniques have been developed for SE detection, not only in microbial culture media, but also in foodstuffs such as cheese, potato salad, ham, and milk [22]. These methods increase the sensitivity of the ELISA procedure by either: (A) the addition of an avidin-biotin complex to detect either SEA or SEB, or (B) inclusion of avidin polyclonal antibodies against SEB together with a biotin-streptavidin amplification system. ELISA-based methods have also been developed for the detection of leukocidin in milk samples [60] originating from cows suffering from mastitis; leukocidin was detected in milk samples containing a concentration as low as 30 ng/mL [46]. ELISAs are currently commonly used for SE detection; they have the advantage of being very specific and highly sensitive, but the procedure requires a long incubation period and is difficult to adapt for multi-SE detection. In addition, the optical immunoassays developed are either based on chemiluminescence or electrochemiluminescence, or surface-enhanced Raman scattering (SERS); the most commonly used optical immunoassays for SEs detection is the fluorescent immunoassay.

Other techniques developed for the detection of SEs, include fluorescence resonance energy transfer (FRET)-based immunoassays and time-resolved fluorescence (TRF) assays [34]. The detection limit for SEA is 0.09 ng/mL when using ELFA, while the VIDAS system cannot detect *S. aureus* enterotoxins at concentrations under 0.25 ng/mL [50]. In addition, due to the fact that multiple dyes and fluorescent nanoparticles can be used as labels, fluorescence immunoassays are very well suited for simultaneous detection of multiple SEs; in fact, a diagnostic detection system was developed using this method, which allows simultaneous detection of staphylococcal enterotoxins SEA, SEB, SEC1, SED, SEE, SEG, and SEI [34].

Electrochemical immunoassays were also developed for SE detection, as is the case for SEC1; these techniques are simple, sensitive, portable, cheap, and reproducible. In addition, a variation of the above procedure, known as sandwich electrochemical immunoassay, allows SEB detection. The sensitivity of these methods involving antigen/antibody interaction, can be increased by signal amplification using a variety of labeling methods. These immunological procedures were further developed in a method known as scanning electrochemical microscopy (SECM)-based immunoassay, reported to detect leukocidin toxin as a concentration of 5.25 pg/mL [58].

Mass-based immunoassays rely on measuring small mass variations; one such an example is the piezoelectric crystal immunosensor used to detect SEB. In this model, the specificity is provided by polyclonal antibodies against the SEB protein, while the antigen binding to the antibody provides an increase in the mass attached to the quartz crystal, hence allowing SEB quantification [34]. Yet another method developed for SE detection involves immunomagnetic separation, requiring magnetic beads coated with specific antibodies, in combination with MALDI-TOF MS (matrix-assisted laser desorption ionization-time of flight mass spectrometry) [50].

Antibody-based biosensors are relatively expensive and entail the difficulties associated with antibody purification and modification [51]; these caveats make antibody-based biosensors expensive and, hence, not suitable for regular sample-testing procedures [50].

### 4.4. New Trends in Staphylococcus aureus Toxin Identification: Aptamers, Molecularly Imprinted Polymers, Proteomics, and Next-Generation Sequencing

Novel methodologies, developed in the last few years for toxin detection in food, include aptamers, mass spectrometry (MS)-based methods, next-generation sequencing, and molecularly imprinted polymers (MIPs).

Aptamers are short single-stranded nucleic acid chains (DNA or RNA) that specifically recognize and bind to target molecules; they are synthesized in vitro by a combinatorial chemical technology known as SELEX (systematic evolution of ligands by exponential enrichment). Aptamers bind their target molecules with a similar affinity and specificity as antibodies. Aptamers, however, have a series of advantages over antibodies: (A) they are easy to produce, by chemical synthesis; (B) have long half-lives; (C) their molecular weights are small; (D) their high stability makes them resistant to both denaturation and renaturation; (E) they are non-toxic; (F) are stable under a wide-range of pH values, temperatures, and ionic environments; and (G) they possess a structural memory, which allows them to rapidly refold into their original structure. In addition, aptamers bind to their complementary strand, generating a double-stranded DNA molecule that can be amplified by PCR, thus creating triple helix structures that can be digested by exonucleases [61]. A wide variety of aptamers have been developed for the detection of either *S. aureus* cells or their toxins (SEA, SEC1, SEB, α toxin), as well as the identifications of proteins such as peptidoglycan, teichoic acid, and protein A [61]. Moreover, an optical-based biosensor that uses an aptamer-modified sandwich ELISA assay has been constructed for the detection of the α toxin secreted by *S. aureus* [61]. Fluorescence resonance energy transfer (FRET)-based biosensors can detect the SEA protein in milk, at a concentration of 8.7 ng/mL; while inorganic fluorescent reporters, such as graphene oxide (GO)-based optical biosensors, can detect *S. aureus* cells at a concentration of 8 cfu/mL, and SEC1 at 6 ng/mL, the latter representing the highest sensitivity for a toxin [61]. This method provides the simultaneous detection of multiplex SEs (SEA, SEB, and SEC1); it uses multicolor lanthanide-doped TRF-labeled aptamers as bioprobes and graphene oxide (GO) as the resonance energy acceptor. In addition, peptide aptamers, designed from phage-peptide libraries, are currently used as novel recognition molecules in biosensor technology [34].

Molecularly imprinted polymers (MIPs) are synthetic polymers constructed by the molecular imprinting technique, which leaves cavities in the polymer matrix that display affinity for a particular template. MIPs are a key methodology to design biosensors capable of detecting the presence of microorganisms in foodstuffs; although the technique is mainly used in food chemistry procedures, such as enzyme tracking or the analysis of organoleptic properties. A potentiometric sensor based on an MIP was designed for the specific diagnosis of staphylococcal exotoxin A; the advantage of this technique is that the tests can be carried out using a simple potentiometer [62].

Proteomic-based methods can be applied for the direct detection of toxins in complex samples; they display high accuracy, reproducibility, and sensitivity. MALDI-TOF MS (matrix-assisted laser desorption ionization time-of-flight mass spectrometry) and liquid chromatography coupled to tandem mass spectrometry (LC-MS/MS) are the main mass spectrometry techniques applied to pathogen detection in foodstuffs. MALDI-TOF MS is used for peptide pattern “fingerprint” identification; this pattern is specific for each individual microorganism, while the individual peaks obtained in the MS analysis represent a specific peptide or protein. On the other hand, LC-MS/MS is commonly used for peptide/protein sequencing, peptide/protein identification and/or quantification, and peptide/protein biomarker monitoring [63,64]. Routine pathogen identification can be performed by MALDI-TOF MS; they are based on the fingerprint displayed by intracellular proteins, which is identified by comparing the data obtained to that of reference strains deposited in a variety of databases available. A variety of studies have evaluated the effectiveness of the MALDI-TOF MS technique in the assessment of extracellular pathogenic factors; this procedure was considered a promising approach, due to its low cost and ease of use [65]. Tonacini and coworkers successfully used MALDI-TOF MS to identify the SEB toxin in the culture media of an *S. aureus* strain isolated from food; SEB identification was confirmed by comparison with purified SEB protein (28.2 kDa), as well as by amino acid sequence analysis. It is of great concern, that the SEB protein was found not to be affected by either pasteurization or a variety of cooking procedures [65]. Bittar and coworkers described the use of MALDI-TOF MS, followed by software analysis, to develop a reliable model to accurately identify PVL in *S. aureus* strains [66]. Furthermore, *S. aureus* delta-toxin production was successfully evaluated by whole cell MALDI-TOF MS analysis, during a MS-mediated microbiological identification procedure [67]. In addition, biomolecular interaction analysis mass spectrometry (BIA-MS) can detect bacterial toxins in food samples; this technology applied surface plasmon resonance (SPR) to detect the binding of toxins to antibodies, previously immobilized on the surface of a sensor chip, followed by MALDI-TOF MS analysis to identify the bound toxins. Using this technique, Nedelkov and coworkers identified the SEB protein, present in either milk or mushroom samples at a concentration of 1 ng/mL [68].

The LC-ESI-MS/MS method requires SE proteins to be treated with proteases, the resulting peptides are then separated by liquid chromatography (LC), followed by electrospray tandem mass spectrometry (ESI-MS/MS) analysis, to obtain either the molecular weight of the SE proteins or their primary amino acid sequences. The LC-ESI-MS/MS technique successfully detected SEA and SEB, in either juices, milk, green beans, crackers, chicken meat, or shrimp, at a concentration of 3 pmol/mL. The advantages of this method over traditional techniques are two-fold, it can detect smaller amounts of toxin, and the toxin assay can be carried out directly in the foodstuffs, without requiring a previous purification step. However, this method also has a caveat, as foodstuffs with high protein content produce a large number of peptides that suppress the electrospray [34,47,48]. Whole cell analysis can also be performed by LC-ESI-MS/MS; Carrera and coworkers identified the presence of toxins and other virulence factors (including LukF-PV and mazF) in foodborne strains of *S. aureus* [69]. *S. aureus* strains involved in mastitis were also analyzed to detect the presence of peptides originating from phages [70]; and the LC-ESI-MS/MS method was also used to detect toxins, proteins of phage origin, antimicrobials, and other virulence factors, with the aim of characterizing and identifying different *Streptococcus* species associated with mastitis [70,71].

Next-generation sequencing (NGS) is another technique applied to detect pathogens and their specific genes. *S. aureus* isolates were collected from nasal samples in cattle, pigs, goats, and sheep, as well as from the farm workers taking care of the animals, followed by whole-genome sequencing analyses using the Illumina MiSeq Platform. The results indicated the presence of *Tsst* and SEs genes in some of the bacterial isolates, however, the PVL gene was not found in any of the isolates [72]. Targeted metagenomics and whole genome sequencing could be useful techniques in the future, for rapid toxin or gene identification.

## 5. Toxins in *S. aureus* Treatment

Currently, multidrug-resistant *S. aureus* infections can only be treated with antibiotics such as vancomycin and linezolid. Therefore, it is essential to develop alternative techniques to combat these bacteria, including vaccines and therapeutic antibodies capable of controlling the growth of *S. aureus*, as an alternative to discovering novel antibiotics [8]. Abril et al. analyzed virulence factor peptides present in *S. aureus* and *S. uberis*, including toxin peptides, by LC-ESI-MS/MS, and concluded that these peptides represent putative targets for vaccines aimed to prevent bovine mastitis [71]. Chang and coworkers tested the ability of a recombinant staphylococcal enterotoxin type C mutant vaccine to prevent an experimental bovine infection caused by a *S. aureus* strain. High antibody titer against SEC was confirmed in all vaccinated individual [73]. Vaccines based on LukS-PV and LukF-PV toxins were demonstrated to confer protection in a mouse model for bacteremia [74]. Furthermore, vaccination of ewes with partially purified PVL, contaminated with α-hemolysin, conferred partial protection against the mastitis disease produced by strains of *S. aureus* [19]; while Hla antisera was reported to inhibit the toxic effects of *S. aureus*, by reducing bacterial adhesion to the bovine mammary epithelial cell layer and increasing neutrophil populations, thus reducing the microbiota present in milk [10].

The use of toxins in the production of vaccines, to induce humoral immunity and generate antibodies against *S. aureus*, could represent a successful way of reducing the invasivity of infections. This approach could neutralize virulence factors, immune evasion molecules, and surface factors, targeting them for destruction [8]. This preventive approach could represent a useful treatment in the amelioration of mastitis infections produced by *S. aureus* [24].

## 6. Conclusions

The aim of this review is to provide a more detailed understanding of the host–*S. aureus* interactions that play a role in the development of mastitis in ruminants, with a particular emphasis on the action of bacterial toxins on host tissues. In addition, it summarizes and explains the common and novel techniques used to identify *S. aureus* exotoxins in foodstuffs. More studies are still needed to improve our understanding of many aspects of staphylococcal toxin biology, as well as their ability to cause food poisoning (SFP) outbreaks. Knowledge in this area is advancing steadily, but further research is essential, not only to further characterize *S. aureus* toxins and improve the diagnosis, but also to develop novel therapeutics, to replace or potentiate current disease treatments (that heavily rely on antibiotics) and prevent the deleterious effects of this bacterium on both animal and human health, as well as avert the high economic losses, produced by *S. aureus* and its toxins, on the dairy industry.

## Figures and Tables

**Table 1:** Principal *S. aureus* exotoxins involved in food poisoning.

Toxin	Principal Toxins in Food Poisoning	Gene	Activity
Toxic shock syndrome toxin 1 (TSST-1)	TSST-1	*tst*	superantigen activity
Staphylococcal enterotoxins (SE)	SEA, SEB, SEC1, SEC2, SEC3, to SEE. SEG to SER, and SEU	*sea* to *see*, *seg* to *ser*, and *seu*	superantigen activity
SE-like toxins	SEG, SEH, SEI, SER, SES, SEIY, and SET	*seg*, *seh*, *sei*, *ser*, *ses*, *seiy*, and *set*	superantigen activity. Without emetic properties or have not been tested yet
Leukocidins	Panton-Valentine leukocidin (PVL), LukPQ, LukMF’, LukAB, and LukED	*lukPV*, *lukPQ*, *lukM*, *lukA* and *lukB* genes, and *lukED*	pore-forming toxins
Hemolysins	α hemolysin (Hla) and β hemolysin (Hlb)	*hla* and *hlb.*	pore-forming toxins
Exfoliative toxins (ETs)	ETA to ETE	*eta* to *ete*	serine proteases that specifically cleave Dsg1 *

* desmoglein 1 (Dsg1).

**Table 2:** Commercial kits available for the detection of staphylococcal enterotoxins in foodstuffs.

Test Kit	Manufacturer	Foods Covered	Features
VIDAS Staph enterotoxin (SET) Immunoassay	BioMérieux	Dairy products, meat, and seafood, etc.	N/A
VIDAS Staph enterotoxin II (SET2) Immunoassay	BioMérieux	Milk and milk products, canned foods, dehydrated foods, meat, seafood, and shellfish, etc.	Detects SEA-SEE in 80 min, providing accurate results within the same day.
TECRA Staphylococcal Enterotoxin VIA	3M Microbiology Canned	Canned mushrooms, nonfat dry milk, canned lobster bisque, beef, and pasta, cooked chicken, and cheese	Fast, reliably detects SEA, SEB, SEC1, SEC2, SEC3, SED, and SEE from food, food-related products, and enrichment cultures with a sensitivity of 1 ng/mL
RIDASCREEN Immunoassay	R-Biopharm, Darmstadt, Germany	Various, including cheese	Sandwich enzyme immunoassay for the identification of SETs A, B, C, D, E in fluids and solid foods, as well as in bacterial cultures
Transia (Transiatube and TransiaPlate) Immunoaffinity, ELISA	Diffchamb, Lyon, France	Milk and dairy products	N/A
SET-RPLA	Oxoid	A wide variety of food and food products such as dairy, meat, and meat products	Detects staphylococcal enterotoxins in a wide variety of foods, providing a semiquantitative result. Sensitivity of the test is 1 ng/mL of extract
SET-RPLA “SEIKEN” RPLA	Denka Seiken		RPLA test employing separately sensitized with highly specific antibodies for SEs A, B, C, and D. Results are semiquantitative

Abbreviations: N/A, not applicable; RPLA, reversed passive latex agglutination assay; SEs, staphylococcal enterotoxins; SEA, staphylococcal enterotoxin A; SEB, staphylococcal enterotoxin B; SEE, staphylococcal enterotoxin E; SETs, staphylococcal enterotoxins. Table adapted and modified from Reddy et al., 2017 [8], under Creative Commons Attribution 4.0 International License.
